# Stress Cardiomyopathy (Takotsubo Cardiomyopathy)

**DOI:** 10.4137/cmc.s3324

**Published:** 2009-09-17

**Authors:** Samer Khouri, Naser Imran

**Affiliations:** Division of Cardiovascular Medicine, University of Toledo, Toledo, Ohio, USA.

**Keywords:** stress cardiomyopathy, physical stress, emotional stress

## Abstract

**Background:**

Due to the rise in the number of reports of stress cardiomyopathy in the literature, awareness of this condition is increasing. Although different names have been used to describe this condition, the similarities in clinical, electrocardiographic, echocardiographic, and angiographic features suggest that they represent the same spectrum of diseases with different underlying causes. The pathophysiology of stress cardiomyopathy remains controversial.

**Methods:**

We describe a series of four cases of stress cardiomyopathy admitted to our institution over a period of six months with different presentations, but similar clinical course, EKG, echocardiographic, and catheterization findings. The ages ranged from 22 to 81 years; all four females. All showed characteristic wall motion abnormalities by imaging in the absence of significant coronary artery disease, with spontaneous recovery of left ventricular function with conservative therapy.

**Results:**

Although the patients presented with different clinical scenarios, all four showed characteristic features of stress cardiomyopathy suggesting that the pathophysiology affecting the myocardium was the same. We present a review of the literature with a discussion of the history of this condition, characteristic clinical features, and diagnostic criteria used in the past as well as the suggested pathophysiology of this condition.

**Conclusion:**

Stress cardiomyopathy is an underdiagnosed reversible cardiomyopathy triggered by severe emotional or physical stress. It represents a spectrum of conditions with reversible severe left ventricular systolic dysfunction that includes neurogenic cardiomyopathy. It is not confined to the Japanese population and can affect people of any ethnic background or nationality.

## Introduction

Stress cardiomyopathy is one of the few reversible cardiomyopathies. First described in 1990, awareness and recognition of this condition is increasing. We report here on a series of four cases that presented to our institution within a period of six months.

A review of the literature reveals characteristic clinical, echocardiographic, and angiographic features that overlap with neurogenic cardiomyopathy, which leads one to believe that these are not separate entities, but are representations of the same spectrum of disease with different underlying causes.

## Case 1

**Figure 1.**
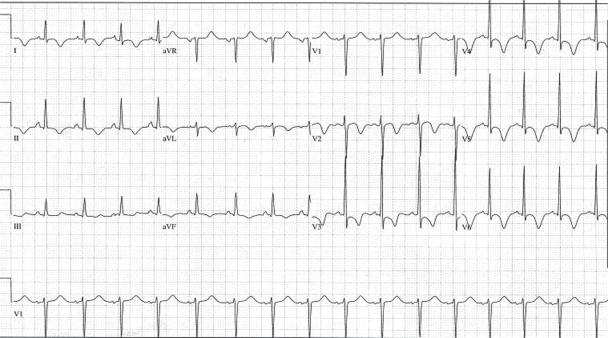
EKG showing deep T wave inversions across precordial leads in a patient with stress cardiomyopathy.

**Figure 2.**
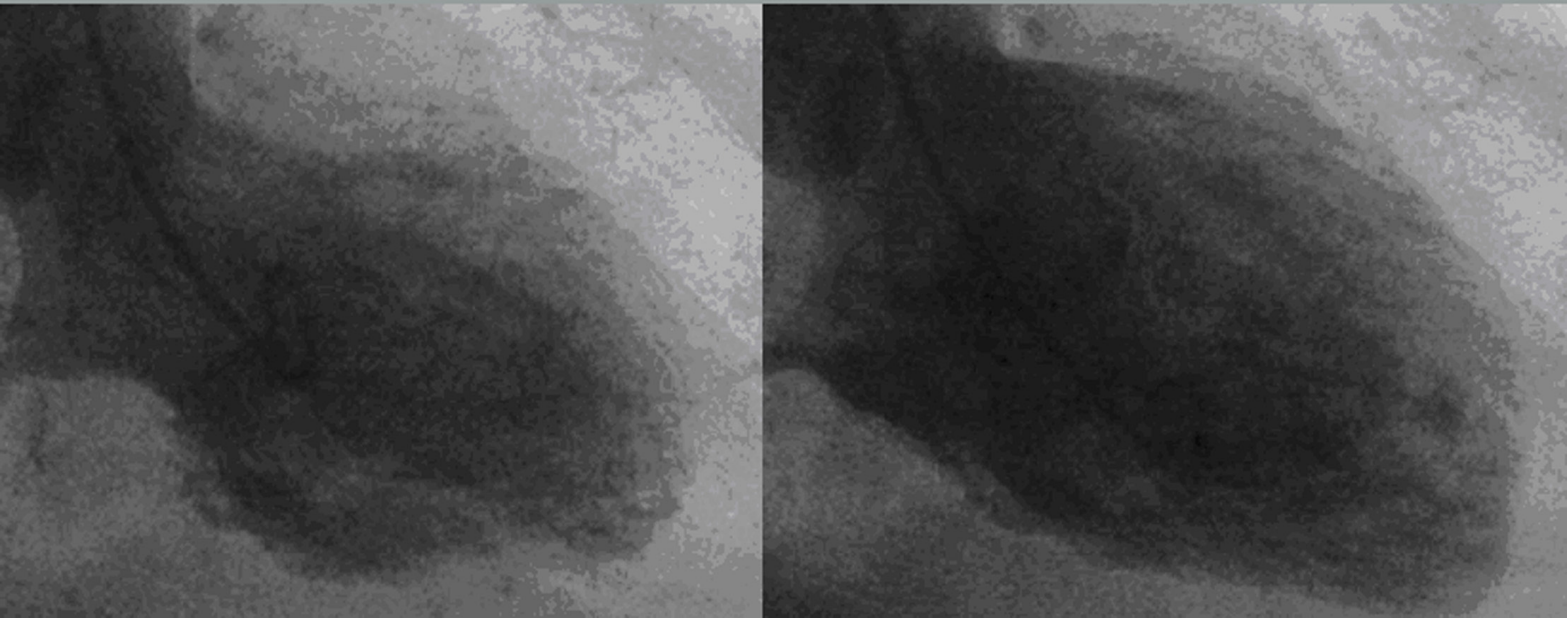
Systole and diastole during left ventriculography showing severe hyokinesis of the apical two thirds of the ventricle and preservation of systolic function in the basal segments.

A 57-year-old, white female with a history of longstanding rheumatoid arthritis, hypertension, and fibromyalgia was admitted six weeks after kyphoplasty with a complaint of severe anterior chest pain, odynophagia, and generalized weakness. Physical examination was remarkable only for diffuse joint deformities and oral candidiasis. EKG on admission showed diffuse T wave inversion (Fig. 1) in the precordial leads, and cardiac enzymes were slightly elevated with a peak troponin of 1.1 (normal range for our institution 0.00–0.05 ng/mL). Diagnosed with acute non-ST elevation myocardial infarction, a cardiac catheterization study was performed which showed mild nonobstructive atherosclerotic disease of the left anterior descending and left circumflex coronary arteries. A left ventriculogram showed hypokinetic middle segments with an akinetic apex (Fig. 2). The basal segments appeared to be normal. Echocardiography confirmed the wall motion abnormalities and an ejection fraction of approximately 40%.

She was treated with ACE inhibitors and continued antihypertensive therapy. Further investigations revealed the presence of severe erosive esophagitis secondary to candida albicans, which was treated with antifungal agents prior to discharge home.

She returned to cardiology clinic for follow-up about six weeks later and repeat echocardiography showed normal left ventricular systolic function with no regional wall motion abnormality.

## Case 2

A 57-year-old lady with a known history of chronic obstructive pulmonary disease (COPD), on home oxygen, mild pulmonary hypertension, and history of anemia, was brought to the ER with complaints of increased shortness of breath due to COPD exacerbation. The patient required intubation due to respiratory failure. It was noted that her ECG showed diffuse ST-T wave abnormalities and cardiac enzymes demonstrated an increased troponin level up to 0.87 ng/mL. She had a heart catheterization that showed an ejection fraction of 10% with normal epicardial coronary arteries. Echocardiography confirmed this finding with severe hypokinesis in the mid and apical segments of the left ventricle. Repeated echocardiogram at one month showed normal global left ventricular systolic function with normal ejection fraction of 60% with no wall motion abnormalities.

## Case 3

An 81-year-old, hypertensive and diabetic, African-American female was admitted to the surgical service with intractable nausea and vomiting. She was diagnosed with gallstone pancreatitis. Shortly after administration of intravenous cefazolin, she developed an acute allergic reaction manifesting as wheezing, dyspnea, and hypertensive urgency. Over the next 12 hours, she progressed to respiratory distress, requiring intubation and ventilatory support.

EKG showed ST elevations in precordial leads V2 to V6. Cardiac enzymes showed a peak troponin of 1.47 ng/mL. A cardiac catheterization was performed which showed mild nonobstructive coronary artery disease. Left ventriculogram showed basal hyperkinesis with apical and anterior wall hypokinesia. Echocardiogram performed four days later revealed normal systolic function with no regional wall motion abnormality. She remained hemodynamically stable throughout her stay and was discharged home after a laparoscopic cholecystectomy three weeks later.

## Case 4

A 22-year-old, white female with a history of adult polycystic kidney disease, hypertension and depression was admitted to the neurosurgery service from a community hospital where she presented with severe sudden onset headache and had been diagnosed with subarachnoid hemorrhage. Diffuse T wave inversions developed in the precordial leads on the EKG over the next 24 hours and troponin elevation to 4.89 was noted. Echocardiography revealed severely reduced ejection fraction of 28% with akinesis of the distal two thirds of the ventricle and basal hyperkinesis. Although the EKG remained abnormal, she remained hemodynamically stable and denied any significant chest symptoms. She appeared to improve from the neurological standpoint over the next four days.

Unfortunately, she developed another subarachnoid hemorrhage two days later, which resulted in her death. Repeat echo on the day of her death, prior to removal of life support, showed return of cardiac function to normal with an ejection fraction of 56% and no regional wall motion abnormality.

## Discussion

### Definition and prevalence

Stress cardiomyopathy is a severe but reversible form of left ventricle dysfunction that mimics acute myocardial infarction in its presentation, with the absence of any obstructive coronary artery disease.

In the large majority of cases, ballooning of the akinetic apical segments of the left ventricle is seen. Pavin et al reported first case from France.^1^ Following this, Sato et al described the appearance of the affected ventricle as resembling an octopus trap used by Japanese fishermen, hence the term “Tako-tsubo” (Tako meaning octopus and tsubo meaning pot).^2^ Many other terms have been used to describe this condition including apical ballooning syndrome, stress induced cardiomyopathy, broken heart syndrome, and ampulla cardiomyopathy (Table 1). Since the original report, variations have been described affecting the basal segments and not involving the apex.

Originally thought to mainly affect the Japanese population, cases have since been reported from the USA, Brazil, Germany, Belgium,^3^ Italy,^4^ and Australia among others, disproving the myth that the condition is confined to the Japanese or Caucasian population. The largest reported case series, not surprisingly, was from Japan.

Although the true incidence of stress cardiomyopathy is unknown, it has been reported to represent approximately 1.2%–2.2% of cases admitted as acute myocardial infarctions in the USA.^5^,^6^ We believe that it remains underreported and goes unrecognized in many centers, especially when patients present with non-cardiac symptoms.

### Clinical presentation

Stress cardiomyopathy is characterized by severe reversible systolic dysfunction typically affecting the apical and mid segments of the myocardium. The majority of reported cases have been postmenopausal females. Patients present with severe chest pain and/or dyspnea. EKG shows ST segment elevation in the precordial leads with gradual resolution of ST elevation and/or inversion of T waves in most cases. Tachy-arrhythmia, like ventricular tachycardia and ventricular fibrillation, might be the initial presentation, and death can occur in rare cases.^7^ Cardiac enzyme markers for infarction are typically minimally elevated. Echocardiography reveals moderate to severe mid ventricular hypokinesis, akinesis, and ballooning of the apex with normal or hyperkinetic basal segments.^7^–^10^ Also, patients might have severe hypotension and cardiogenic shock that may require intra-aortic balloon pump (IABP).^7^ Villareal et al. reported left ventricle outflow tract (LVOT) obstruction due to hyperdynamic basal segments that caused severe mitral regurgitation and might contributed to the development of shock.^11^ LVOT obstruction was also reported during dobutamine infusion.^10^ In addition, apical thrombus and stroke have been reported.^12^ Cardiac catheterization reveals normal epicardial coronary arteries or mild coronary artery disease that fails to explain the degree and distribution of wall motion abnormality. In the vast majority of cases, systolic function improves to normal over the next few days to weeks with appropriate management (Table 2). Recurrences are uncommon, but have been reported.^13^

### Pathophysiology

The pathophysiology of stress cardiomyopathy remains unclear. Many hypotheses are considered, which include catecholamine-triggered injury and endothelial dysfunction which have many supportive data. Epicardial coronary spasm and obstruction of the left ventricle outflow tract have minimal and sporadic data and are becoming less viable.

Cases of myocardial stunning in patients with large subarachnoid hemorrhages^14^,^15^ have in the past been attributed to excess sympathetic activity and massive surges of catecholamines affecting the myocardium. In the recently reported case series by Wittstein et al, plasma catecholamine levels in 13 patients with stress cardiomyopathy were compared to levels in seven patients with Killip class III myocardial infarction.^7^ It was shown that catecholamine levels were 2 to 4 times higher in patients with stress cardiomyopathy compared to patients with myocardial infarction, again suggesting a central role of exaggerated sympathetic stimulation in the mechanism underlying stress cardiomyopathy. Furthermore, Mori et al showed increased reactivity to sympathetic stimulation in apical segments of the left ventricle.^16^ These findings support the proposed mechanism of the direct effect of catecholamines on the myocardium, suggesting either a “differential distribution of catecholamine receptors in the myocardium” such that the apex has more receptors than mid-segments and that the base is devoid of receptors;^17^ or a “variation in responsiveness of receptors” with apical receptors being more susceptible to effects of the catecholamine surge.^16^

If catecholamines are indeed the cause of myocardial dysfunction, the question then arises as to how they cause stunning of the myocardium. It has been proposed that catecholamine induced spasm of the epicardial coronary arteries or dysfunction of the coronary microcirculation^18^ may play a role. Spasm of multiple coronary arteries was demonstrated in approximately 15% of cases from Japan, but not demonstrated in cases reported from the rest of the world.^19^ If the effect is at the level of the microcirculation, the differential response from base to apex remains unexplained.

These findings of catecholamine cardiotoxicity might lead to high intracellular calcium concentration and it was proposed that overload of calcium might lead to cardiac dysfunction.^20^–^22^ In general, this hypothesis does not explain why there are segmental abnormalities in stress cardiomyopathy rather than global hypokinesis.^23^ However, gender differences due to adrenergic agonists response might explain in part female prevalence of this syndrome.^16^,^20^,^21^

In the case series from Minneapolis-St. Paul by Sharkey et al, cardiac magnetic resonance (CMR) assessments showed the absence of delayed Gadolinium hyperenhancement, a finding usually seen in patients with myocardial scar or infarction, in 21 out of the 22 patients with stress cardiomyopathy.^7^ This suggests that the myocardium is probably stunned, but remains viable during this time, in keeping with the excellent prognosis and usual return of normal systolic function over the ensuing few weeks. These finding were consistent with other investigators’ findings who performed CMR on patients with stress cardiomyopathy where the wall motion abnormalities were diffuse and in more than one coronary artery distribution.^2^,^19^ Furthermore, CMR was pivotal in demonstrating the absence of myocardial infarction, myocarditis, necrosis, loss of membrane activities and scar formation.^10^ Endomyocardial biopsy of patients with stress cardiomyopathy shows lymphocytic interstitial infiltrates and/or contraction band necrosis as opposed to the polymorphonuclear inflammatory infiltrate seen in myocardial infarction.^7^ A similar histological appearance is seen in patients with cardiomyopathy secondary to subarachnoid hemorrhage, also thought to be caused by excess catecholamines.^7^ People dying from close range gunshot wounds to the head and other forms of violent assault, when examined post-mortem, were also found to have similar myocardial histologies, again suggesting a link between severe emotional stress, excess catecholamines and stress cardiomyopathy.^24^ Pretreatment with alpha and beta adrenoreceptor blockers has been shown to prevent apical ballooning in a rat model of emotional stress.^25^

Endothelial dysfunction has been proposed as one of the features of stress cardiomyopathy as well.^26^ Bybee et al^9^ and Kuriso et al^27^ demonstrated increased TIMI frame counts in all three coronary vessels on coronary angiography of patients with stress cardiomyopathy, lending further credibility to this hypothesis.

Feola et al, studied the myocardial blood flow (MBF) and coronary flow reserve (CFR) by use of positron emission tomography (PET) scan at presentation and after three months.^28^ Three patients underwent rest-stress adenosine perfusion with nitrogen-13 ammonia and metabolism study with fluorine-18 fluorodeoxyglucose PET scan. The images showed the impairment of tissue metabolism in the dysfunctional left ventricular segments in the acute phase, mainly in the apical segments and progressively less in the medium segments, which normalized three months later. The quantitative analysis of MBF and CFR showed a reduction in the acute phase in apical segments in comparison to basal segments that recovered completely after three months.

The criteria proposed by Bybee et al from the Mayo Clinic for the clinical diagnosis of stress cardiomyopathy, excludes patients with intracranial bleeding, pheochromocytoma, and recent significant head trauma.^8^ However, the pattern of myocardial dysfunction unexplained by coronary artery anatomy, associated severe emotional and physiological stress, and the probable role of excess catecholamines in both conditions would suggest that these conditions are also part of the spectrum of stress cardiomyopathy and not separate entities.^2^,^23^,^29^ In addition, intracranial pathology can produce the same histopathology findings seen in stress cardiomyopathy,^30^ and cardiac sympathectomy prevents brain-mediated cardiac injury.^31^

In conclusion, we proposed to revise the Mayo Clinic diagnosis criteria by omitting the last criteria that exclude the pheochromocytoma and neurogenic pathology (Table 3). In our series, an acute physical stress is the common culprit that was associated with stress cardiomyopathy rather than emotional stress.

## Conclusion

Stress cardiomyopathy represents a spectrum of conditions manifesting as reversible severe systolic dysfunction, with characteristic EKG, echo, and angiographic findings. It is not confined to the Japanese or Caucasian population, and may affect patients of any ethnicity. Cardiomyopathy secondary to severe neurological events like subarachnoid hemorrhage, status epilepticus, or intracranial trauma is probably a manifestation of the same condition and not a separate entity. The true incidence of stress cardiomyopathy is probably higher than currently estimated, as it remains unrecognized and under-reported in many centers. Although the exact mechanism of stress cardiomyopathy remains not fully understood, it appears that the activation of the sympathetic nervous system, resulting in a surge of catecholamines due to severe emotional or acute physical disease, probably plays a central role in the pathology of this condition.

## Figures and Tables

**Table 1.** Synonyms for stress cardiomyopathy.

Takotsubo’s cardiomyopathy Apical ballooning syndrome Broken heart syndrome Ampulla cardiomyopathy Stress induced cardiomyopathy Neurogenic myocardial stunning

**Table 2.** Classical features of stress cardiomyopathy.

Female predominance Acute onset of symptoms Pathology triggered by any form of severe emotional or physical stress Myocardial dysfunction affecting distribution of more than one coronary artery usually apical and midsegments Lack of angiographic evidence of obstructive coronary artery disease or plaque rupture Mild elevation of cardiac biomarkers ST segment elevation or T wave inversion on EKG Reversal of myocardial dysfunction over several weeks

**Table 3.** Clinical criteria for diagnosis of stress cardiomyopathy, revised from Bybee et al.^18^

Transient akinesis or dyskinesis of the left ventricular apical and mid-ventricular segments Absence of obstructive coronary disease or angiographic evidence of acute plaque rupture New electrocardiographic abnormalities (either ST-segment elevation or T-wave inversion) Absence of myocarditis
